# The Effects of Post-Traumatic Stress Disorder on Memory Function

**DOI:** 10.1192/j.eurpsy.2026.11874

**Published:** 2026-07-31

**Authors:** M.-A. Panagopoulou

**Affiliations:** Faculty of Medicine, University of Patras, Patras, Greece

## Abstract

**Introduction:**

Post-traumatic stress disorder (PTSD) is a mental health condition that develops after exposure to a highly traumatic event. It is characterized by re-experiencing, avoidance, hyperarousal, and negative alterations in mood. Emerging evidence indicates that these symptoms frequently co-occur with memory dysfunction. A core feature of PTSD is the duality of memory disturbances: traumatic amnesia, the inability to recall all or part of a traumatic event, and emotional hypermnesia, the vivid, intrusive recall of trauma.

**Objectives:**

This study aims to explore the relationship between PTSD and memory dysfunction.

**Methods:**

A literature review was conducted to examine the relationship between PTSD and memory dysfunction. PubMed was searched using the terms “posttraumatic stress disorder” and “memory.” Articles were selected based on relevance to PTSD and memory outcomes. Key findings regarding the interaction between trauma exposure, memory disturbances, and clinical implications were summarized.

**Results:**

Memory disturbances, including intrusive memories and dissociative amnesia, are central to the clinical presentation of PTSD. While PTSD primarily affects trauma-related memories, individuals may also struggle to recall everyday events, segment ongoing activities, or learn and remember neutral information. Memory alterations can manifest as reduced recall (traumatic amnesia) or exaggerated recall (hypermnesia), including vivid flashbacks and nightmares. Individuals with PTSD often have difficulty retrieving specific autobiographical memories, particularly positive ones, which may contribute to symptom severity. Deficits in episodic and contextual memory, along with failure of extinction learning, underscore the hippocampus’s key role in PTSD. Individuals with PTSD often exhibit bilateral hippocampal atrophy, reflected in reduced hippocampal volume on neuroimaging. Trauma-related hippocampal reduction may result from the hippocampus’s high sensitivity to stress hormones, particularly glucocorticoids and adrenal steroids. Beyond the hippocampus, the amygdala is another subcortical structure critically involved in PTSD, especially in mediating intrusive and hyperarousal symptoms. The amygdala plays a central role in fear conditioning and the encoding of fear memories. Although structural changes are uncommon, neuroimaging studies consistently demonstrate amygdala hyperactivation in response to trauma-related or negative emotional stimuli. This heightened activation may underlie the hallmark symptoms of re-experiencing and hypervigilance observed in PTSD. Finally, functional imaging studies reveal disrupted connectivity among the hippocampus, amygdala, and prefrontal cortex, contributing to impaired contextual processing, emotional dysregulation, and the persistence of maladaptive fear memories.

**Conclusions:**

Understanding the mechanisms that cause memory dysfunction in PTSD may guide targeted therapeutic interventions.

**Disclosure of Interest:**

None Declared

